# Supplementary figures and images for: PPARγ/mTOR Regulates the Synthesis and Release of Prostaglandins in Ovine Trophoblast Cells in Early Pregnancy
Source: Vet Sci. 2022 Nov 21;9(11):649. doi: 10.3390/vetsci9110649 (PMC9694237; doi:10.3390/vetsci9110649)

Figure S1

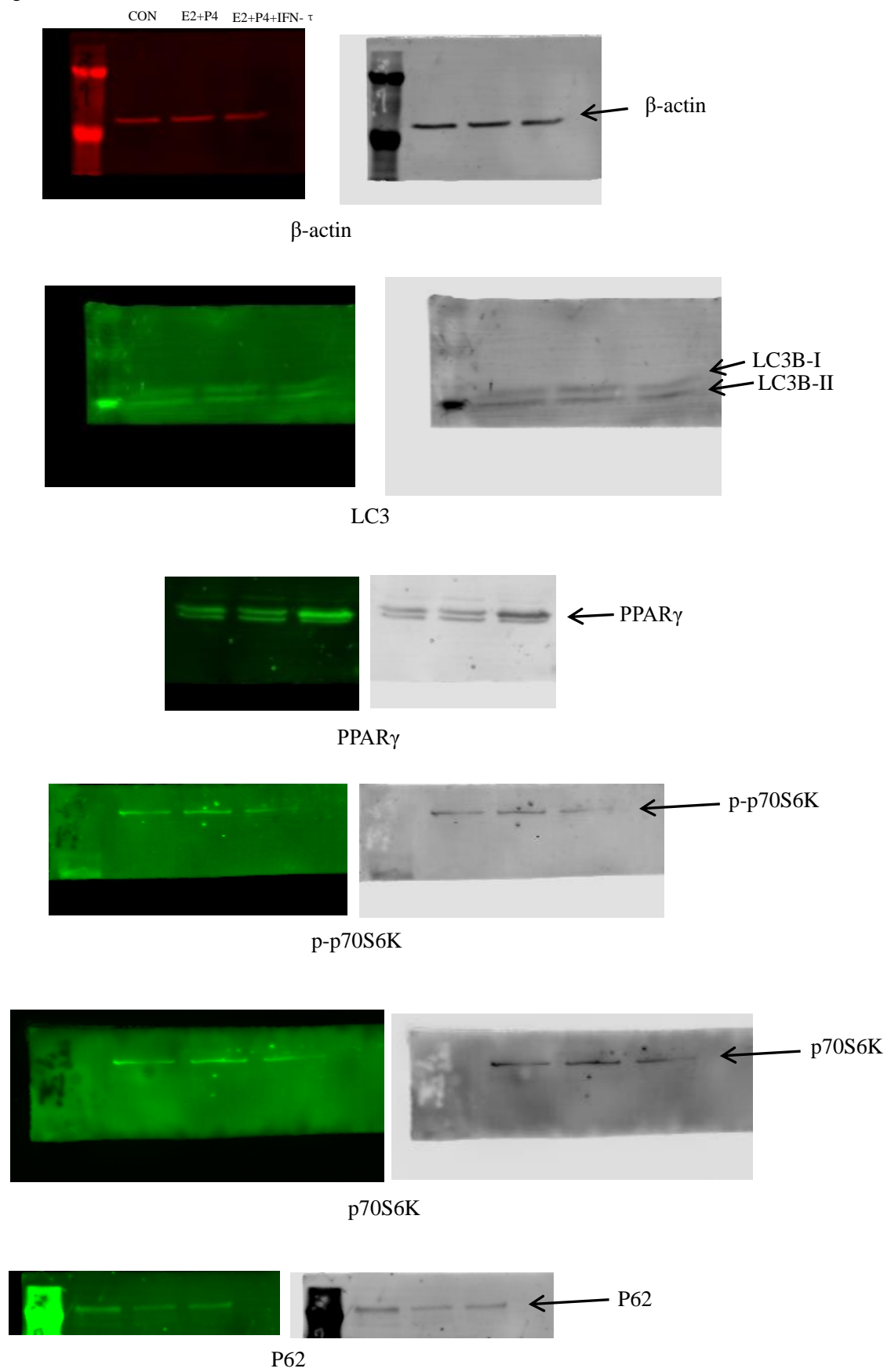

Figure S2

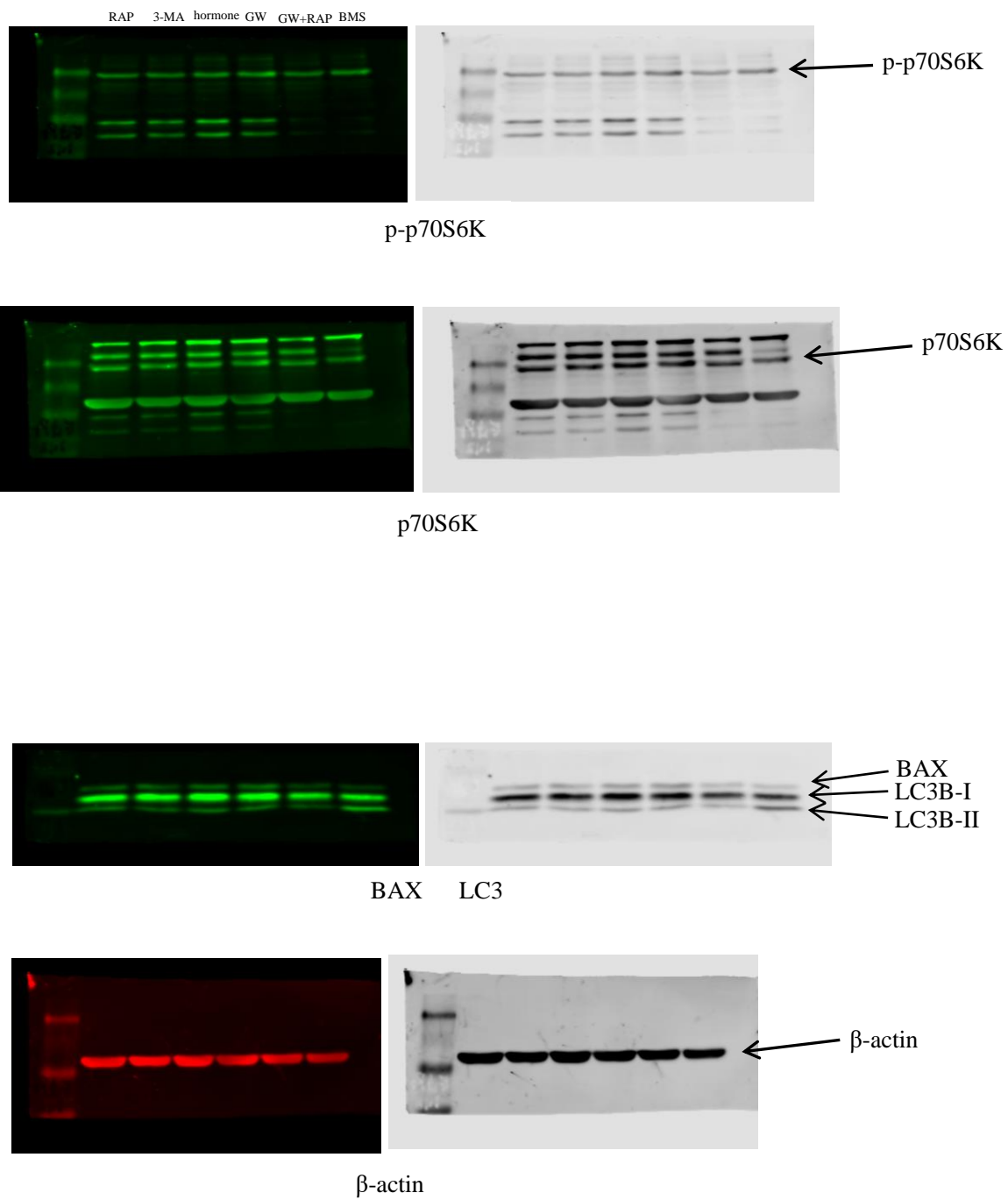

Supplement: Supplementary file 1 [file vetsci-09-00649-s001.zip › vetsci-1995905-supplementary.pdf]
